# Protocols for Adventitious Regeneration of *Amelanchier alnifolia* var. *cusickii* and *Lonicera kamtschatica* ‘Jugana’

**DOI:** 10.3390/plants10061155

**Published:** 2021-06-06

**Authors:** Júlia Hunková, Monika Szabóová, Alena Gajdošová

**Affiliations:** Plant Science and Biodiversity Center, Institute of Plant Genetics and Biotechnology, Slovak Academy of Sciences, 950 07 Nitra, Slovakia; julia.hunkova@savba.sk (J.H.); m.szaboova@savba.sk (M.S.)

**Keywords:** in vitro adventitious regeneration, shoot proliferation, *Amelanchier alnifolia*, *Lonicera kamtschatica*

## Abstract

The aim of this work was to assess the regeneration capacity of *Amelanchier alnifolia* var. *cusickii* and *Lonicera kamtschatica* cv. ‘Jugana’ from different types of explants under various hormonal treatments. The whole leaves, petioles, and internodal segments of in vitro plants were examined as explants. Several plant growth regulators (cytokinins and auxins) were evaluated for their ability to induce adventitious regeneration. Direct and indirect organogenesis was achieved under certain culture conditions in both species. The frequency of shoot regeneration was strongly dependent on concentrations of plant growth regulators in the induction media (*L.* *kamtschatica* ‘Jugana’) or concentrations of plant growth regulators in the induction media and type of explant (*A. alnifolia* var. *cusickii*). Results showed that leaves were not suitable explants for *A. alnifolia* var. *cusickii*. Both species were able to regenerate shoots from internodal segments and petioles. The highest induction of shoots was obtained on Murashige and Skoog (MS) medium enriched with 2 mg/L thidiazuron (TDZ) and 0.5 mg/L indole-3-butyric acid (IBA) for *Amelanchier alnifolia* and with 1 mg/L TDZ and 0.2 mg/L indole-3-acetic acid (IAA) for *L.* *kamtschatica* ‘Jugana’. Obtained adventitious shoots were further proliferated in order to investigate their multiplication capacity. The multiplication of shoots was successful in all cultivars, with the best results reported in *A. alnifolia* var. *cusickii* (7.07 shoots/explant on average).

## 1. Introduction

Berries belong to the group of economically important crops mostly due to their benefits on human health [1]. It is known that many non-traditional small fruit species are being recognized for their content of biologically active compounds and serve as a valuable and natural source of vitamins, antioxidants, anthocyanins, and polyphenols with strong health-promotion effect [2,3,4,5]. *Amelanchier alnifolia* Nutt. (saskatoon berry) is a fruit bearing shrub native to the Canadian prairies and southern and northern territories of the United States. This berry is potentially attractive because of its frost-resistance, disease-resistance, and decorative purposes [6], moreover also of its high yields and fruit quality, which is very similar to blueberry. Saskatoon fruit could be a suitable supplement for modern human nutrition, as the berries are rich source of polyphenols, flavonoids (gallic acid, rutin, quercetin), and anthocyanines [7]. *Lonicera kamschatica* (honeysuckle) is a deciduous shrub native to Russia and Japan. Similarly to saskatoon, it is frost- and disease-resistant and can be grown in lowlands as well as mountain regions [8]. This species has dark-blue, elongated-shape fruits, which contain a high amount of vitamin C, anthocyanins, and flavonoids (quercetin, rutin) [9,10].

The demand for small fruit is continuously growing, especially in the food industry for a variety of products [11]. Both saskatoon and honeysuckle berries can be eaten fresh or dried, or be processed to other products like jam, syrup, desserts, or vines [12,13]. In order to fulfill the increasing demand for healthy planting material, tissue culture techniques are being used as an alternative to traditional propagation methods. They are less time-demanding and in case of adventitious regeneration they can also serve as a prerequisite for the transformation approaches used in genetic improvement of selected cultivars [14,15]. However, plant regeneration in vitro depends on such factors as species, genotype, and explant. Environmental conditions (culture medium, plant growth regulators) also significantly influence the rate of regeneration [16]. Thus, there is still a need for development of new or optimization of existing procedures, especially for less commercialized cultivars and species.

Reports about adventitious regeneration in *Lonicera* spp. are rather limited. Shoot induction was achieved from leaf explants in *Lonicera nitida, Lonicera japonica and Lonicera macranthoides* [17,18,19], stem segments in *Lonicera tatarica* [20], or calli cultures in *Lonicera japonica* [21]. The combinations of cytokinins 6-benzylaminopurine (BAP), thidiazuron (TDZ), and kinetin (KIN) with different auxins were used in a wide range of concentrations. However, due to some inconsistencies, no general recommendations can be made for *Lonicera* spp. as each protocol needs to be modified to the individual species. No study has been published yet dealing with adventitious regeneration of *L. kamtschatica* ‘Jugana’ and the same is also valid for *A. alnifolia* var. *cusickii*.

The research presented in this study was undertaken because of a total absence of relevant data for these two species. The primary goal was to examine adventitious plant regeneration in saskatoon and honeysuckle using three different types of tissues. The effect of different combinations of plant growth regulators (PGR) for callogenesis and organogenesis, shoot induction and multiplication were evaluated. The reported results clarify factors controlling adventitious shoot regeneration of *A. alnifolia* var. *cusickii* and *L. kamtschatica* ‘Jugana’ and provide rapid protocols allowing further plant multiplication and improvements by in vitro techniques and genetic engineering.

## 2. Results

### 2.1. Amelanchier alnifolia var. cusickii Shoot Induction and Multiplication

**Figure 1:**
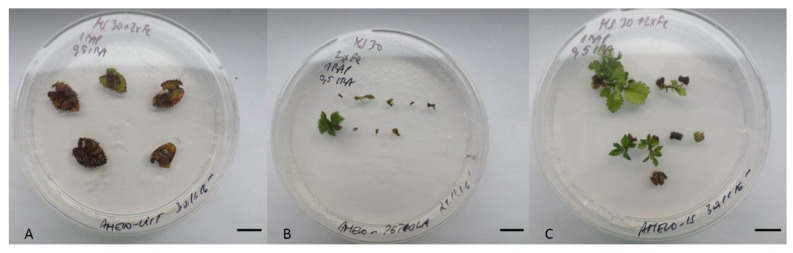
*A. alnifolia* adventitious shoot induction from leaves (**A**), petioles (**B**), and internodal segments (**C**) on induction media. Explants after eight weeks of cultivation on MS medium with 1 mg/L BAP and 0.5 mg/L IBA. The length of the scale bar is 1 cm.

Testing of explant type showed that *A. alnifolia* var. *cusickii* leaves of in vitro plants were not suitable for adventitious shoot induction. The same outcome was observed with petioles as the majority of them had necrotized shortly after being placed on an induction medium. The significantly highest induction of shoots was recorded after using internodal segments (IS) as explants (Table 1, Figure 1).

The type of explant and type of cytokinin (BAP vs. TDZ) was a statistically significant factor for callogenesis and shoot induction, while the type of cytokinin was not significant for the number of induced adventitious shoots. Under the influence of BAP, no calli emerged and only direct regeneration was observed. With the use of TDZ, shoots originated both directly and indirectly. The highest rate of induction was observed after cultivation on MS medium with 2 mg/L TDZ and 0.5 mg/L IBA. It is worth mentioning that TDZ in concentrations higher than 2 mg/L led more frequently to shortened stems with longer, occasionally deformed leaves, while no such traits were observed under the influence of BAP.

ANOVA suggests a significant effect (*p* ≤ 0.05) of explant type on the callus formation, shoot induction, and number of induced adventitious shoots. Plant growth regulator was a significant factor for callus formation and also shoot induction but did not affect the number of shoots. The interaction of both factors was significant for shoot induction as well as their number (Table 2). The mean number of shoots induced on media with BAP or TDZ reached 3.58 and 3.22, respectively. The average number of shoots per explant type was 0.17 (leaf), 1.33 (petiole), and 4.05 (IS) (Supplementary Table S1).

**Figure 2:**
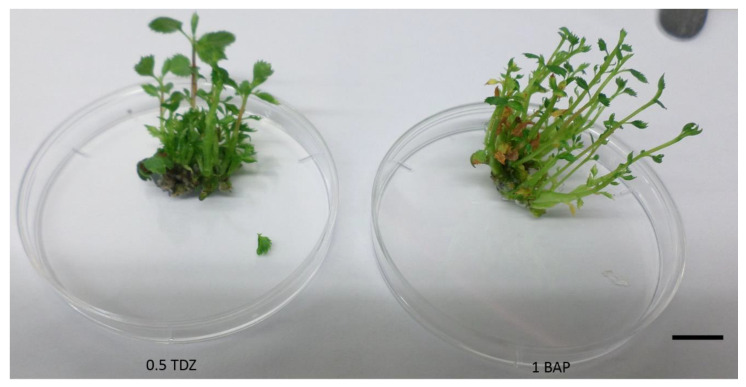
Effect of 0.5 mg/L TDZ and 1 mg/L BAP in combination with 0.5 mg/L IBA on *A. alnifolia* var. *cusickii* multiplication. Adventitious shoots after four weeks of cultivation on MS medium. The length of the scale bar is 1 cm.

Both cytokinins were examined during multiplication phase to evaluate whether prolonged influence of TDZ will result in worsened shoot growth. Multiplication index of shoots reached 7.07 shoots/explant for BAP and 6.57 shoots/explant for TDZ. The mean number of shoots evaluated after each subculture did not rise significantly under the influence of TDZ (6.93, 6.13, and 6.67, respectively). In the presence of BAP, the highest mean number of shoots was recorded after 1st subculture, followed by a significant decrease in the next two subcultures (10.57, 4.57, and 6.10, respectively) (Figure 2).

### 2.2. Lonicera Kamtschatica ‘Jugana’ Shoot Induction and Multiplication

**Figure 3:**
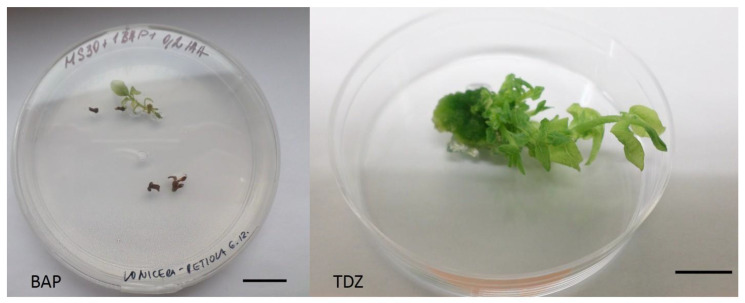
Adventitious shoots of *L. kamtschatica* ´Jugana´ after eight weeks of cultivation on MS medium with 0.2 mg/L IAA, 1 mg/L BAP, and 1 mg/L TDZ. The length of the scale bar is 1 cm.

The organogenesis efficiency in *L. kamtschatica* ´Jugana´ was rather low compared to *A. alnifolia* var. *cusickii* (Supplementary Table S2). The highest rate of induction was observed when petioles were used, internodal segments were also suitable explants. The use of leaves resulted in their necrosis. Significant differences were observed in callogenesis and shoot organogenesis depending on the hormone type (Table 3). Shoots induced from explants cultivated on media with BAP were chlorotic, very fragile, and thin, without active growth. They perished shortly after transferring on multiplication medium. The same result was observed also on medium containing both BAP and kinetin (data not evaluated). On the contrary, shoots induced under the influence of TDZ had thicker stems with larger leaves and occasional chlorosis (Figure 3).

They originated only indirectly from green, proliferating calli and were actively growing and elongating during the whole induction phase. The significantly highest number of regenerated shoots was recorded after cultivation on MS medium with 1 mg/L TDZ and 0.2 mg/L IAA.

**Figure 4:**
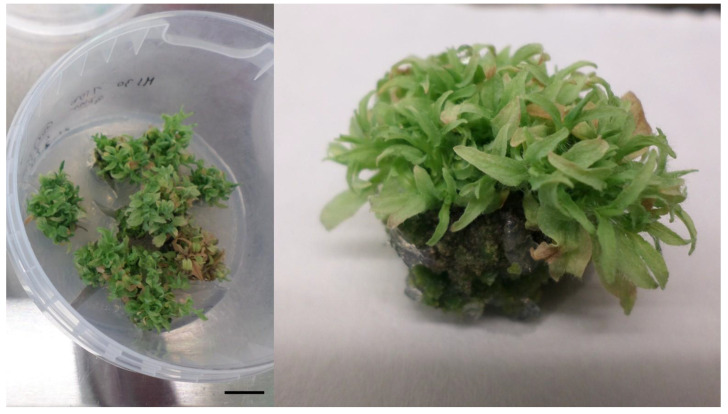
Adventitious shoots of *L. kamtschatica* ´Jugana´ after four weeks of cultivation on MS medium supplemented with 36.7 mg/L FeNaEDTA, 1 mg/L TDZ and 0.2 mg/L IAA. The length of the scale bar is 1 cm.

ANOVA suggests a highly significant (*p* ≤ 0.01) effect only of PGR treatment on callus formation, shoot induction and the number of induced adventitious shoots (Table 4). The mean number of shoots cultivated on media with BAP or TDZ reached 0.36 and 3.07, respectively. The average number of shoots per explant type was 0.29 (leaf), 1.64 (petiole), and 2.82 (IS) (Supplementary Table S2). Multiplication of shoots was successful under the influence of 1 mg/L TDZ and 0.2 mg/L IAA on MS medium supplemented with 36.7 mg/L FeNaEDTA to avoid shoot chlorosis. Most of the shoots multiplied formed big clumps with a massive callus on the base part but their elongation was reduced (Figure 4). The average multiplication index after three months of cultivation reached 7.03 shoots/explant.

## 3. Discussion

According to our knowledge, no study was reported concerning adventitious regeneration of *Amelanchier* spp. so far, therefore comparison of our results is possible only with related members of Rosaceae family. Mahmood and Hassanein [22] achieved 36.7% regeneration of adventitious shoots from leaf discs of *Rosa hybrida* ‘Eiffel Tower’ cultivated on modified MS medium with 0.5 mg/L BAP, 1 mg/L GA_3_, and 0.5 mg/L NAA. Thidiazuron in the concentration of 1 mg/L did not result in shoot induction but increasing its concentration led to a higher rate of callogenesis which was observed also in our study. Reports were published concerning regeneration from other types of explants, such as internode sections [23], cotyledons [24], and petioles [25]. Shoot induction was not observed from *Prunus avium* leaves on MS medium with 2 mg/L TDZ and 0.5 mg/L IBA. The regeneration was confirmed only on QL (Quoirin and Lepoivre) medium [26] with the same growth regulators [23]. As in our study, the internode sections as explants resulted in more effective shoot formation in almost all cultivars. Feyissa et al. [25] observed that TDZ was superior to BAP in callus induction and shoot regeneration from leaves of *Hagenia abyssinica*. Previous studies on *H. abyssinica* showed that low concentration of TDZ (<0.2 mg/L) enhanced direct shoot regeneration while high concentrations promoted callus induction and shoot regeneration. However, high concentration of TDZ led to shoot elongation inhibition [25], what confirms findings in our research. The regeneration rate was improved significantly only after the increase of BAP above 5 mg/L which might indicate that concentration in our study was too low for successful induction. According to Vujović et al. [27], a sudden decrease in the multiplication index can be caused by repeated subcultivation as it was observed for two *Prunus* spp. cultivars. This might be a possible explanation because the mother plants in this experiment were already cultivated for at least one year. The multiplication index (7.1 shoots/explant) in this study was comparable with the results already published for shoots of *A. alnifolia* var. *cusickii* which originated from axillary buds [28].

As far we know, no protocol has been published for adventitious regeneration of *L. kamtschatica* ´Jugana´ yet. Leaves and stem internodes were used as explants in existing studies with other honeysuckle species [18,19,20]. Leaves of *L. japonica* ‘Hall’s Profilic’ cultivated on ½ LS (Linsmaier and Skoog) medium [29] with 1 or 3 mg/L BAP were the most responsive explants in the work of Georges et al. [18] (48–53%, respectively), while TDZ resulted in no induction. This does not coincide with our results where TDZ was significantly better cytokinin compared to BAP. However, the species-dependent response might alter the outcome of the experiment. Differentiation of shoots from callus cultures of *L. japonica* Thunb. was evaluated by Hui et al. [21]. The best results (5.50 shoots/explant) were obtained after cultivation on WPM medium with 0.5 mg/L BAP and 1.5 mg/L NAA. Moreover, NAA resulted in significantly higher differentiation than IAA. This supports the findings in our study about PGR treatment directly influencing the induction of shoots. The multiplication index (7.0 shoots/explant) achieved in our study was comparable with the results already published for *L. kamtschatica* (Sevast.) Pojark shoots propagated from axillary buds [8]. According to our previous experiences with *Amaranthus* spp. adventitious regeneration and shoot multiplication [30], we suppose that reduced elongation growth of shoot clumps formed on medium with 1 mg/L TDZ and 0.2 mg/L IAA could be stimulated by their transfer on medium with 1 mg/L BAP, as was also reported by Van Le et al. [31]. Fira et al. [32] reported that *L. kamtschatica* represent a challenge for micropropagation due to a relatively low proliferation rates in some cases, shorts shoots, physiological problems like necrosis of the apices and hyperdricity on media with high BAP concentration, which confirms our findings.

In conclusion, this study presents protocols for adventitious regeneration of two economically important small fruit species. As far as is known, this study is the first describing adventitious shoot induction and proliferation of saskatoon (*A. alnifolia* var. *cusickii*) and honeysuckle (*L. kamtschatica* ´Jugana´). Recommendations presented herein could be applied to related species or used for further genetic improvement of selected cultivars via transformation or other biotechnology techniques.

## 4. Materials and Methods

### 4.1. Plant Material

In vitro cultures of *Amelanchier alnifolia* var. *cusickii*, *Lonicera kamschatica* ´Jugana´ were established according to the procedure by Hunková et al. [27,33,34]. Sprouts bearing several axillary or apical buds were taken from mature plants, cut into smaller pieces (1–1.5 cm), and sterilized with 70% (*v/v*) ethanol for 2 min followed by immersion in 0.1% (*w/v*) HgCl_2_ with Tween for 5 min along with three rinses in sterile distilled water.

### 4.2. Shoot Initiation and Multiplication

Single-node explants were placed vertically in sterile Petri dishes (6 cm in diameter) filled with the culture medium. In vitro cultures of *Amelanchier alnifolia* var. *cusickii* and *Lonicera kamschatica* ´Jugana´ were established at MS (Murashige and Skoog) medium supplemented with 30 g/L sucrose (Slavus, Bratislava, SK) and 8 g/L plant agar (Duchefa Biochemie, Haarlem, NL). Medium pH was adjusted to 5.6 before autoclaving 20 min at 1 kg cm^3^ and 121 °C. Growth regulators for shoot initiation were used as follows: 1 mg/L BAP and 0.5 mg/L IBA for *A. alnifolia* var. *cusickii*; 2 mg/L BAP and 0.2 mg/L IAA for *L. kamtschatica* ´Jugana´. After six weeks, 10–15 shoots from each cultivar were transferred to Combiness vessels (Microbox Combiness, Nevele, BE) containing multiplication medium. All plant growth regulators were filter-sterilized before being added to the culture medium. The concentration of plant growth regulators followed the earlier published protocols [27,35,36]. Proliferating shoots were subcultured every 4 weeks. All cultures were maintained in a growth chamber at 22 ± 2 °C with 16 h light period using cool white fluorescent light at a photosynthetic photon flux density 50 μM/m^2^s.

### 4.3. Adventitious Shoot Regeneration

Explants were taken from the upper third part of the shoots two weeks after the last subculture from cultures that had been cultivated for one year. Three types of explants were chosen to compare their ability to induce organogenesis, e.g., whole leaves, petioles, and internodal segments (IS). Initial explants were fully developed parts of the plantlet (no buds etc.). The leaf had been cut together with the petiole, then the petiole was separated and both parts (leaf, petiole) were used whole as individual initial explants. All leaves were cut transversally three-times. Explants were placed horizontally and abaxially on the induction medium and subjected to treatments with various plant growth regulators (Table 5). From each explant type, 25 pieces were used per treatment in one repetition. After eight weeks of induction phase, all explants were examined, and the number of shoots was calculated for each explant individually. The number of shoots was used for statistical evaluation. Following the induction phase, isolated adventitious shoots were transferred to the multiplication medium (Table 5). Due to problems with chlorosis, 36.7 mg/L of FeNaEDTA was added to MS medium for *L. kamtschatica* ´Jugana´. The number of multiplied shoots from 30 randomly chosen explants was recorded after each subculture, once every four weeks. The duration of the experiment was 12 weeks (with three subcultures of the shoots on the fresh culture medium in 4-week interval). Multiplication index was calculated as the amount of shoots/number of explants ratio and indicated the total average number of adventitious shoots per explant.

### 4.4. Statistical Analysis

Statistical analysis of the obtained data were performed by using Statistica 10 software (StatSoft Inc., Tulsa, OK, USA). Analysis of variance (ANOVA) and Fisher LSD test were employed to identify significant differences among the different processes at *p* ≤ 0.05.

## Figures and Tables

**Table 1:** Effect of plant growth regulators (PGR) treatments and explant type on adventitious shoot regeneration of *A*. *alnifolia* var. *cusickii*.

PGR Combinations (mg/L)	Shoot Induction			Regenerated Shoots		
	L	P	IS	L	P	IS
1 BAP + 0.5 IBA	0.000 e	0.040 de	0.160 cd	0.000 c	0.280 c	0.240 c
2 BAP + 0.5 IBA	0.000 e	0.040 de	0.040 de	0.000 c	0.040 c	0.640 bc
3 BAP + 0.5 IBA	0.000 e	0.000 e	0.320 b	0.000 c	0.000 c	1.520 ab
1 TDZ + 0.5 IBA	0.040 de	0.000 e	0.240 bc	0.040 c	0.000 c	0.360 c
2 TDZ + 0.5 IBA	0.000 e	0.000 e	0.680 a	0.000 c	0.000 c	2.120 a
3 TDZ + 0.5 IBA	0.000 e	0.000 e	0.320 b	0.000 c	0.000 c	2.240 a

L—leaves, P—petioles, IS—internodal segments. Means with different letters (a–e) for each group within three columns indicate significant differences by LSD test (*p* ≤ 0.05).

**Table 2:** Summary of two-way ANOVA results for examining the effect of explant and hormone and their interaction of *A. alnifolia* var. *cusickii*.

Effect		Callus Formation		Shoot Induction		Number of Shoots	
	df	F	*p*	F	*p*	F	*P*
explant	2	4.482	0.012 *	61.778	0.000 *	23.249	0.000 *
hormone	5	6.304	0.000 *	5.203	0.000 *	1.961	0.083
explant * hormone	10	1.829	0.054	6.598	0.000 *	2.521	0.005 *
Error	432						

Note: df—degrees of freedom; F—F-value; *p*—probability value; statistical significance at * *p* ≤ 0.05 in the same colum.

**Table 3:** Effect of plant growth regulators (PGR) treatments on callus formation and adventitious shoot regeneration of *L. kamtschatica* ´Jugana´.

PGR Combinations (mg/L)	Callus Formation	Shoot Induction	Regenerated Shoots
1 BAP + 0.2 IAA	0.000 b	0.040 ab	0.040 bc
2 BAP + 0.2 IAA	0.000 b	0.000 b	0.000 c
3 BAP + 0.2 IAA	0.000 b	0.000 b	0.000 c
0.5 BAP + 1.5 KIN + 0.2 IAA	0.000 b	0.027 ab	0.027 bc
1 TDZ + 0.2 IAA	0.133 a	0.080 a	0.360 a
2 TDZ + 0.2 IAA	0.200 a	0.053 ab	0.227 ab
3 TDZ + 0.2 IAA	0.173 a	0.013 b	0.027 bc

Means with different letters (a–c) indicate significant differences by LSD test (*p* ≤ 0.05) in the same column.

**Table 4:** Summary of two-way ANOVA results for examining the effect of explant and hormone and their interaction in *Lonicera kamtschatica* ´Jugana´.

Effect		Callus Formation		Shoot Induction		Number of Shoots	
	df	F	*p*	F	*p*	F	*p*
explant	2	1.002	0.368	2.867	0.058	2.863	0.058
hormone	6	5.518	0.000 *	2.289	0.034 *	3.501	0.002 *
explant * hormone	12	0.562	0.873	1.622	0.082	1.692	0.0653
Error	504						

Note: df—degrees of freedom; F—F-value; *p*—probability value; statistical significance at * *p* ≤ 0.05 in the same column.

**Table 5:** Different plant growth regulators (PGR) treatments for adventitious regeneration of *A. alnifolia* var. *cusickii* and *L. kamtschatica* ´Jugana´.

Species	PGR (in mg/L)	
	Shoot Induction	Shoot Multiplication
A. alnifolia var. cusickii	1–3 BAP + 0.5 IBA	1 BAP + 0.5 IBA
	1–3 TDZ + 0.5 IBA	0.5 TDZ + 0.5 IBA
L. kamtschatica ‘Jugana’	1–3 BAP + 0.2 IAA	1 TDZ + 0.2 IAA
	1–3 TDZ + 0.2 IAA	
	0.5 BAP + 1.5 KIN + 0.2 IAA	

## Supplementary Materials

The following are available online at https://www.mdpi.com/article/10.3390/plants10061155/s1, Table S1: Adventitious shoot induction rate (%) and number of induced shoots from leaves, petioles and internodal segments of *Amelanchier alnifolia* var. *cusickii* grouped according to different explants and planth growth regulators (PGR) treatments, Table S2: Adventitious shoot induction rate (%) and number of induced shoots from leaves, petioles and internodal segments of *Lonicera kamtschatica* ´Jugana´ grouped according to different explants and planth growth regulators (PGR) treatments.

## References

[1] Byrne D.H., Badenes M.R., Byrne D.H. (2012). Trends in fruit breeding. Fruit Breeding.

[2] Moyer R.A., Hummer K.E., Finn C.E., Frei B., Wrolstad R.E. (2002). Anthocyanins, Phenolics, and Antioxidant Capacity in Diverse Small Fruits: Vaccinium, Rubus, andRibes. J. Agric. Food Chem..

[3] Hodges D., Kalt W. (2003). Health functionality of small fruit. Acta Hortic..

[4] Castrejón A.D.R., Eichholz I., Rohn S., Kroh L.W., Huyskens-Keil S. (2008). Phenolic profile and antioxidant activity of highbush blueberry (*Vaccinium corymbosum* L.) during fruit maturation and ripening. Food Chem..

[5] Cuevas-Rodríguez E.O., Yousef G.G., García-Saucedo P.A., López-Medina J., Paredes-López O., Lila M.A. (2010). Characteriza-tion of anthocyanins and proanthocyanidins in wild and domesticated Mexican blackberries (*Rubus* spp.). J. Agric. Food Chem..

[6] Ochmian I., Kubus M., Dobrowolska A. (2013). Description of plants and assessment of chemical properties of three scecies of Amelanchier genus. Dendrobiology.

[7] Rop O., Mlcek J., Jurikova T., Sochor J., Kizek R. (2013). Antioxidant properties od Saskatoon berry (*Amelanchier alnifolia* Nutt.) fruits. Fruits.

[8] Sedlák J., Paprštein F. (2013). Micropropagation of edible honeysuckle. Vědecké Práce Ovocnářské.

[9] Malodobry M., Bienasz M., Dziedzic E. (2010). Evaluation of the yield and some components in the fruit of blue honeysuckle (*Lo-nicera caerulea* ver. Edulis turcz. Freyn.). Folia Hortic..

[10] Jurikova T., Rop O., Mlcek J., Sochor J., Balla S., Szekeres L., Hegedusova A., Hubalek J., Adam V., Kizek R. (2012). Phenolic profile of edible honeysuckle berries (genus *Lonicera*) and their biological effects. Molecules.

[11] Debnath S. (2011). Bioreactors and molecular analysis in berry crop micropropagation—A review. Can. J. Plant Sci..

[12] Zatylny A.M., Ziehl W.D., St-Pierre R.G. (2005). Physiochemical properties of fruit of 16 saskatoon (*Amelanchier alnifolia* Nutt.) cultivars. Can. J. Plant Sci..

[13] Holubec V.K., Štolc K., Paprštein F. (2007). Genetic resources of honeysuckle Lonicera kamtschatica (Sevast.) Pojark., natural condi-tions and variability. Inovace Pěstování Ovocných Plodín.

[14] Meiners J., Schwab M., Szankowski I. (2007). Efficient in vitro regeneration systems for Vaccinium species. Plant Cell Tissue Organ Cult..

[15] Cappelletti R., Sabbadini S., Mezzetti B. (2016). The use of TDZ for the efficient in vitro regeneration and organogenesis of straw-berry and blueberry cultivars. Sci. Hortic.-Amst..

[16] Gahan P.B., George E.F., George E.F., Hall M.A., De Klerk G.-J. (2008). Adventitious regeneration. Plant Propagation by Tissue Culture.

[17] Cambecèdes J., Duron M., Decourtye L. (1992). Interacting Effects of 2,3,5-Triiodobenzoic Acid, 1-Aminocyclopropane-1-Carboxylic Acid, and Silver Nitrate on Adventitious Bud Formation from Leaf Explants of the Shrubby Honeysuckle, Lonicera nitida Wils. ‘Maigrün’. J. Plant Physiol..

[18] Georges D., Chenieux J.C., Ochatt S.J. (1993). Plant regeneration from aged-callus of the woody ornamental species Lonicera japoni-ca cv. “Hall’s Prolific”. Plant Cell Rep..

[19] Wang X., Chen J., Li Y., Nie Q., Li J. (2009). An efficient procedure for regeneration from leaf-derived calluses of Lonicera macran-thoides ‘Jincuilei’, an important medicinal plant. HortScience.

[20] Palacios N., Christou P., Leech M. (2002). Regeneration of Lonicera tatarica plants via adventitious organogenesis from cultured stem explants. Plant Cell Rep..

[21] Hui J.X., Wen S.C., Hua Z.Y., Ming L.X. (2012). Comparative study on different methods for Lonicera japonica Thunb. microprop-agation and acclimatization. J. Med. Plants Res..

[22] Hassanein A.M.A., Mahmoud I.M.A., Ali M.I.M., Ahmed H.A.M. (2018). Essential factors for in vitro regeneration of rose and a protocol for plant regeneration from leaves. Hortic. Sci..

[23] Matt A., Jehle J.A. (2005). In vitro plant regeneration from leaves and internode sections of sweet cherry cultivars (*Prunus avium* L.). Plant Cell Rep..

[24] Canli F., Tian L. (2009). Regeneration of adventitious shoots from mature stored cotyledons of Japanese plum (*Prunus salicina* Lind1). Sci. Hortic..

[25] Feyissa T., Zhu L.H., Negash L., Welander M. (2007). Regeneration and genetic transformation of *Hagenia abyssinica* (Bruce) JF Gmel. (Rosaceae) with rolB gene. PCTOC.

[26] Quoirin M., Lepoivre P. (1977). Improved media for In Vitro culture of *Prunus* spp. Acta Hortic..

[27] Vujović T., Ružić D., Cerović R. (2012). In vitro shoot multiplication as influenced by repeated subculturing of shoots of contem-porary fruit rootstocks. Hortic. Sci..

[28] Hunková J., Libiaková G., Fejér J., Gajdošová A. (2017). Improved Amelanchier alnifolia Nutt. Ex. M. Roem. shoot proliferation by manipulating iron source. Propag. Ornam. Plants.

[29] Linsmaier E.M., Skoog F. (1965). Organic Growth Factor Requirements of Tobacco Tissue Cultures. Physiol. Plant..

[30] Gajdošová A., Libiaková G., Iliev I., Hricová A. (2013). Adventitious shoot induction of *Amaranthus cruentus* L. in vitro. Propag. Ornam. Plants.

[31] Van Le B., My N.D., Jeanneau M., Sadik S., Tu S., Vidal J., Vân K.T.T. (1998). Rapid plant regeneration of a C 4 dicot species: Amaranthus edulis. Plant Sci..

[32] Fira A., Clapa D., Cristea V., Plopa C. (2014). In vitro propagation of Lonicera kamtschatica. Agric. Sci. Pract..

[33] Hunková J., Libiaková G., Fejér J., Vujovic T., Gajdosová A. (2018). Testing of different iron sources and concentrations on shoot multiplication of blackberry (*Rubus fruticosus* L.). Genetika.

[34] Hunková A., Libiaková G., Gajdošová A. Less-known small fruit species and their propagation using in vitro techniques. Proceedings of the Recent advances in neglected and under-utilized species research.

[35] Lloyd G., McCown B. (1980). Commercially-feasible micropropagation of mountain laurel, *Kalmia latifolia*, by use of shoot-tip culture. Comb. Proc. Int. Plant Prop. Soc..

[36] Dziedzic E. (2008). Propagation of blue honeysuckle (*Lonicera caerulea* var. Kamtschatica pojark) in in vitro culture. J. Basic Appl. Sci..

