# Supplementary material for: Protocols for Adventitious Regeneration of Amelanchier alnifolia var. cusickii and Lonicera kamtschatica ‘Jugana’
Source: Plants (Basel). 2021 Jun 6;10(6):1155. doi: 10.3390/plants10061155 (PMC8228500; doi:10.3390/plants10061155)
Supplement: Supplementary file 1 [file plants-10-01155-s001.zip › plants-1169001-supplementary.pdf]

Supplementary Table S1. Adventitious shoot induction rate (%) and number of induced shoots from leaves, petioles and internodal segments of *Amelanchier alnifolia* var. *cusickii* grouped according to different explants and planth growth regulators (PGR) treatments.

	Shoot induction (%)	Induced shoots
**Explant**		
Leaves	0.67^{b}	0.17 ± 0.167
Petioles	1.33^{b}	1.33 ± 1.145
Internodal segments	29.33^{a}	4.05 ± 0.645
**PGR (mg/L)**		
1 BAP + 0.5 IBA	6.67^{b}	2.17 ± 1.01
2 BAP + 0.5 IBA	2.67^{b}	5.67± 5.17
3 BAP + 0.5 IBA	12.00^{b}	3.80 ± 0.99
1 TDZ + 0.5 IBA	9.33^{b}	1.25 ± 0.25
2 TDZ + 0.5 IBA	22.67^{a}	2.95 ± 0.58
3 TDZ + 0.5 IBA	10.67^{b}	5.30 ± 2.20

Different letters (a-b) indicate significant differences by LSD test ($p \leq 0.05$) in the same column. Induced shoots are presented as the means ± standard error.

Supplementary Table S2. Adventitious shoot induction rate (%) and number of induced shoots from leaves, petioles and internodal segments of *Lonicera kamtschatica* ´Jugana´ grouped according to different explants and planth growth regulators (PGR) treatments.

	Shoot induction (%)		Induced shoots
**Explant**			
Leaves	0.57^{a}		0.29 ± 0.29
Petioles	4.57^{a}		1.64 ± 0.61
Internodal segments	4.00^{a}		2.82 ± 0.83
**PGR (mg/L)**		**Leaves**	
1 BAP + 0.2 IAA	4.00^{ab}	BAP	0.00 ± 0.00
2 BAP + 0.2 IAA	0.00^{b}	TDZ	0.67 ± 0.67
3 BAP + 0.2 IAA	0.00^{b}	**Petioles**	
0.5 BAP + 1.5 KIN + 0.2 IAA	2.66^{ab}	BAP	0.67 ± 0.21
1 TDZ + 0.2 IAA	8.00^{a}	TDZ	2.80 ± 1.16
		**Internodal segments**	
2 TDZ + 0.2 IAA	5.33^{ab}	BAP	0.25 ± 0.25
3 TDZ + 0.2 IAA	1.33^{b}	TDZ	4.29 ± 0.89

Different letters (a-b) indicate significant differences by LSD test ($p \leq 0.05$) in the same column. Induced shoots are presented as the means ± standard error.
